# An updated RBD-Fc fusion vaccine booster increases neutralization of SARS-CoV-2 Omicron variants

**DOI:** 10.1038/s41392-022-01185-7

**Published:** 2022-09-17

**Authors:** Deyan Luo, Xiaolan Yang, Tao Li, Nianzhi Ning, Song Jin, Zhuangzhuang Shi, Hongjing Gu, Deyu Li, Yuwei Gao, Hui Wang

**Affiliations:** 1 State Key Laboratory of Pathogen and Biosecurity, Beijing Institute of Microbiology and Epidemiology, Beijing, 100071 China; 2 Key Laboratory of Jilin Province for Zoonosis Prevention and Control, Veterinary Research Institute, Chinese Academy of Agricultural Sciences, Changchun, 130021 China

**Keywords:** Vaccines, Infectious diseases

**Dear Editor,**

Coronavirus disease 2019 (COVID-19) is caused by severe acute respiratory syndrome coronavirus 2 (SARS-CoV-2), which is a novel subset of coronavirus. To this day, the number of confirmed cases is over 500 million with more than 6 million deaths globally. SARS-CoV-2 keeps on evolving into different variants, including Alpha, Beta, Gamma, Delta, and Omicron. From November of 2021, the epidemic strain was Omicron, including B.1.1.529.1 (Omicron BA.1), B.1.1.529.2 (Omicron BA.2), B.1.1.529.2.12.1 (Omicron BA.2.12.1), B.1.1.529.3 (Omicron BA.3), B.1.1.529.4 (Omicron BA.4) and B.1.1.529.5 (Omicron BA.5). Omicron has spread all over the world and become the predominantly strain in most countries, especially Omicron BA.4 and Omicron BA.5. Nevertheless, the Delta variants (B.1.617.2, Delta 617) and some special variants also exist in in the crowd at the same period, such as B.1.640.2 (IHU) which was found in England. Omicron BA.1, Omicron BA.2, Omicron BA.3, Omicron BA.4, and Omicron BA.5 carry more than 30 mutations in the spike (S) protein as well as the receptor binding domain (RBD). These sublineages of Omicron variants share 11 mutations in the RBD region of virus, wherein Omicron BA.1 has 3 unique mutations, Omicron BA.2 has 4 unique mutations, Omicron BA.4 or Omicron BA.5 has 2 unique mutations. The RBD region of Omicron BA.5 is the same as the BA.4.^1^ Given these differences, their antigenic properties cannot be assumed to be the same. Many of these mutations, e.g., K417Y, E484A, N501Y, have been predicted to affect neutralization epitopes.^2^

Indeed, more evidence has shown that the Omicron variants can largely escape from vaccination,^3^ including our RBD-Fc fusion protein vaccine (RBD-Fc-WT Vacc). Currently, this vaccine is at the stage of a multi-regional phase 3 clinical trial. In order to face the mutations of the virus, we developed an update RBD-Fc fusion protein vaccine, containing receptor-binding domain of Omicron BA.1 (RBD-Fc-Omicron Vacc) (Fig. 1a). This variant vaccine was manufactured as a liquid formulation containing 20 μg per 0.5 ml in a vial by ZHONGYIANKE Biotech Co., Ltd., with aluminum hydroxide as the adjuvant. The RBD-Fc fusion vaccine encodes the RBD antigen of SARS-CoV-2 (aa331-524) and human IgG Fc protein as the backbone, which forms a dimeric form of RBD.

**Fig. 1:**
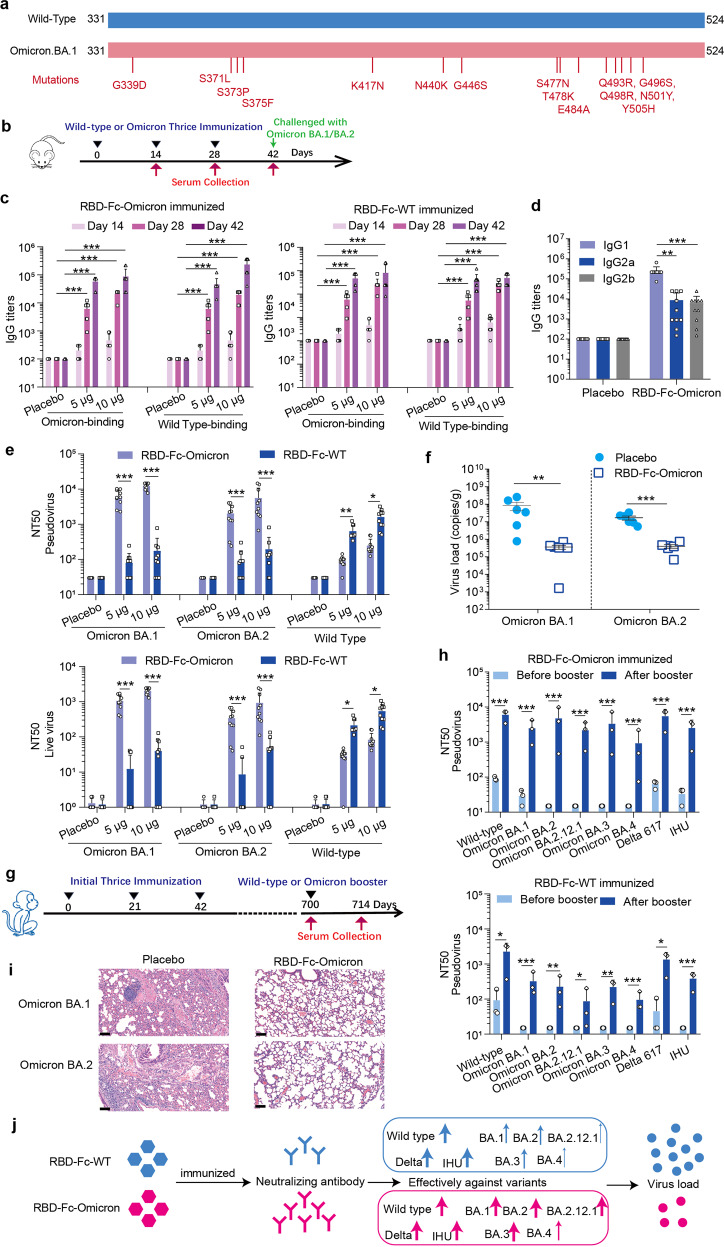
Development and characterization of an updated RBD-Fc fusion protein vaccine against Omicron. **a** Vaccine design, Structural model of amino acid mutations in the RBD of Omicron BA.1 variant of SARS-CoV-2. All mutations in the RBD were shown. **b** The immune program of RBD-Fc-Omicron and RBD-Fc-WT in mice. Groups of female BALB/c mice (*n* = 9-10) were immunized intramuscularly with three doses of 5 μg or 10 μg of RBD-Fc-Omicron at 14-day interval. Sera were collected on day 14, day 28, and 42 after the first immunization. The IgG titers (**c**), antibody isotype (**d**), and the NT50 (**e**) were analyzed. To test the protective effect of RBD-Fc-omicron, BALB/c mice were immunized with RBD-Fc-Omicron Vacc (10 μg/mouse) on day 0, day 14, and day 28. Two weeks after the last immunization, mice were inoculated with Omicron BA.1 and BA.2 intranasally (*n* = 6, 3 × 10^3^ TCID50/mouse), On day 3 post-infection, six mice in each group were sacrificed and harvest. **f** RT–qPCR was used to measure total viral genome copies in lung tissues. **i** Histopathological changes in lung tissues observed by light microscopy using haematoxylin and eosin staining (Scale Bar: 100 μm). **g** The immune program of RBD-Fc-Omicron and RBD-Fc-WT in macaques. Groups of macaques were intramuscularly immunized and boosted with 10 μg vaccines (*n* = 3), and sera were detected on day 700 and day 714 after the first immunization. **h** Neutralizing titers after a homologous or heterologous booster RBD-Fc fusion protein vaccine in macaques. **j** The pattern diagram of our study. The update Omicron vaccine has the immunodominance against VOCs. All data are shown as means ± SD, and a one-way ANOVA with multiple comparisons tests or *t*-test is used (*P <0.05, **P < 0.01, ***P < 0.001)

To evaluate the efficiency of the RBD-Fc-Omicron Vacc, female BALB/c mice were immunized with three dose of aluminum adjuvanted RBD-Fc-Omicron and RBD-Fc-WT (5 µg, 10 µg each mouse) via intramuscular administration on day 0, day 14 and day 28, the aluminum was used as placebo. The sera were collected on day 14, day 28, and day 42 after the first immunization (Fig. 1b). To evaluate the immunogenicity of this variant vaccine, the IgG antibody titers, and antibody isotype were detected by ELISA method firstly, and the 50% neutralizing antibody titers (NT50) against pseudovirus and live virus were also tested. To test the protective effect of RBD-Fc-Omicron, BALB/c mice were immunized and challenged with Omicron variants Omicron BA.1 and Omicron BA.2. A detailed description of the methods is available in the supplementary files. After three doses of immunizations at 14-day interval, high levels of IgG antibodies (Fig. 1c) and neutralizing antibodies were induced by the RBD-Fc-Omicron Vacc at day 14, day 28, and day 42 after the first immunization. Meanwhile, the antibody induced by this variant vaccine can neutralize Omicron BA.1 and Omicron BA.2 epidemic strains (Fig. 1e, Supplementary Fig. S1), suggesting the immunodominance of RBD-Fc-Omicron Vacc currently. We tested both the RBD-WT and RBD-Omicron binding antibodies against all mice immunized two vaccines. We did not find the difference between the RBD-Fc-WT immunized groups and the RBD-Fc-Omicron immunized groups (Fig. 1c). In comparison with RBD-Fc-WT immunized groups, the titers of neutralizing antibody against Omicron BA.1 and Omicron BA.2 (Fig. 1e) are much higher in RBD-Fc-Omicron immunized group. The major antibody isotype present in the sera of RBD-Fc-Omicron immunized mice is IgG1 (Fig. 1d), which suggested that the RBD-Fc-Omicron Vacc could induce Th2-type immune response. We did not detect CD8^+^ T cell responses in vaccinated groups that also support this conclusion (Supplementary Fig. S2). The protective assay displayed that the update vaccine could reduce viral load (Fig. 1f) and pathological lesion (Fig. 1i) in lung significantly.

Qin’s team suggested that booster immunization in mice could increase immunogenicity of vaccine against Omicron Variant of Concern (VOCs) in mouse model.^3^ To make sure the effect of a heterologous or homologous booster of our vaccine, groups of macaques that have received three doses of RBD-Fc-WT Vacc were further boosted with a fourth dose homologous (RBD-Fc-WT Vacc) or heterologous vaccine (RBD-Fc-Omicron Vacc) at day 700 post prime immunization. The sera were collected on day 700 and day 714 to evaluate the cross-immune reactivities against wild-type virus, three Omicron VOCs, Delta variant, and IHU (Fig. 1g). Our results showed that a homologous booster immunization induced neutralizing antibody production against all VOCs, and the geometric mean titers (GMTs) against wild-type (WT), Omicron BA.1 and Omicron BA.2, Omicron BA.2.12.1, Omicron BA.3, Omicron BA.4, Delta 617 and IHU variants increased to 1558.9, 270.7, 162.9, 51.8, 183.7, 83.9, 1094.6 and 337.7 compared with control. This homologous booster immunization induced lower antibody production against Omicron variants that may because of the unique mutations in virus strain (Fig. 1h). A heterologous booster immunization induced high levels neutralizing antibody production, and the GMTs against WT, Omicron BA.1, and Omicron BA.2, Omicron BA.2.12.1, Omicron BA.3, Omicron BA.4, Delta 617 and IHU variants increased to 5651.5, 2031.0, 2611.2, 1680.1, 1926.7, 430.7,4652.5 and 1888.2. The heterologous vaccine booster could induce higher levels of neutralizing antibody titers against WT, Omicron VOCs, Delta variant, and IHU variants than that induced by homologous vaccine booster (Fig. 1h). It is about 16-fold higher against Omicron BA.2, 32-fold higher against Omicron BA.2.12.1, 10-fold higher against Omicron BA.3 and about 5-fold higher against other variants than macaques followed a homologous vaccine booster. Since the heterologous vaccine booster could induce higher levels neutralizing antibody titers against WT, Omicron VOCs, Delta variant and IHU variants, the booster of a new RBD-Fc fusion protein vaccine that directly targets the Omicron BA.1 RBD will be benefit to stop Omicron spread all over the world (Fig. 1j).

We designed this Omicron update vaccine against SARS-CoV-2 for the first time, and evaluated the serum across activities against epidemic strains of mice and macaques. The update RBD-Fc-WT Vacc will be another choice for further vaccine immune program. Particularly, we boosted macaques with our update vaccine almost after two years, which could display the importance of basic immunization with RBD-Fc-WT Vacc. Our data presented here suggested that a fourth dose immunization would lead to an increasement in neutralizing antibodies not only against the wild-type SARS-CoV-2 but also the Omicron variants and some other variants, even though the basic immunization was performed a long time ago. Both homologous and heterologous booster vaccinations with our subunit vaccine represent a rational strategy in response to the Omicron emergency, especially the heterologous booster vaccination. Based on these differences of gene mutations, these Omicron variants antigenic properties cannot be assumed to be the same. But our results still showed the cross-reactivity of these Omicron variants. As RBD-Fc-Omicron was produced under the same procedure and release specification as its original version, which was published in 2021.^4^ The immunogenicity of this first RBD-Fc-Omicron vaccine candidate has been evaluated in animal models and should be tested in clinical trials next step.

## Supplementary information

Supplementary materials

## Data Availability

All data are available in this article, supplementary materials, or available from the corresponding authors.
