# Supplementary material for: An updated RBD-Fc fusion vaccine booster increases neutralization of SARS-CoV-2 Omicron variants
Source: Signal Transduct Target Ther. 2022 Sep 17;7:327. doi: 10.1038/s41392-022-01185-7 (PMC9482636; doi:10.1038/s41392-022-01185-7)
Supplement: Supplementary file 1 — Supplementary materials [file 41392_2022_1185_MOESM1_ESM.docx]

Supplementary Materials for

An updated RBD-Fc fusion vaccine booster increases neutralization of SARS-CoV-2 Omicron variants

Deyan Luo, Xiaolan Yang, Tao Li, Nianzhi Ning, Song Jin, Zhuangzhuang Shi, Hongjing Gu, Deyu Li, Yuwei Gao, Hui Wang

Correspondence to: [geno0109@vip.sina.com](mailto:geno0109@vip.sina.com) or gaoyuwei@gmail.com.

**This PDF file includes:**

Materials and Methods

Figures. S1 to S2

Materials and Methods

Construction and purification of RBD-Fc-Omicron Vacc

The coding sequence for RBD-Fc-Omicron region, which fused residues 331-524 from the spike protein of the SARS-CoV-2 (B.1.1.529.1) and human IgG1-Fc together, were synthesized by Beijing JOINN Bilogic Co., Ltd. (Beijing, China). For protein expression, the plasmid was first transfected into CHO-K1 cells and then stable clones were isolated by the limiting dilution method in the presence of 50 μM of Methionine Sulfoximine. The cell culture supernatants were collected and analyzed by western blot analysis using a commercial antibody (Sino Biological Inc. Beijing, China) against SARS-CoV-2 RBD. The fusion protein was purified with Gel/Ion chromatography. The vaccine was manufactured according to current Good Manufacturing Practice by ZHONGYIANKE Biotech Co., Ltd. (Tianjin, China), a liquid formulation containing 20 μg or 40 μg per 0.5 ml in a vial, with aluminum hydroxide as the adjuvant. The purity of drug substance was tested using High Pressure Liquid Chromatography and SDS-PAGE. The stability of drug product was tested in different conditions. Vaccines were stored at 2 ℃ to 8 ℃ before use.

Animals, cells and vaccine candidate

BALB/c mice were purchased from Weitonglihua Company (Beijing, China). All animal procedures were reviewed and approved by the Animal Experiment Committee of Laboratory Animal Center, Beijing Institute of Microbiology and Epidemiology and Changchun Veterinary Research Institute, China (Assurance Number: IACUC-DWZX-2020-028). Vero cell (ATCC, CCL-81) was maintained in Dulbecco’s minimal essential medium supplemented with 10% fetal bovine serum and penicillin (100 U/ml)-streptomycin (100 µg/ml) (Thermo Fisher Scientific, Waltham, USA). An update RBD-Fc fusion protein vaccine, containing receptor-binding domain of Omicron BA.1 (virus strain B.1.1.529.1, RBD-Fc-Omicron) was developed. CHO-K1 cell (ATCC, CCL-61) was kept in ZHONGYIANKE Biotech Co., Ltd. (Tianjin, China), and Huh7 cell (M-C1078) was kept in our lab.

Three-year old Macaca fascicularis (n **=** 3 each group) was immunized with three doses of SARS-CoV-2 RBD-Fc-WT (10 µg each animal), and then boosted with RBD-Fc-WT or RBD-Fc-Omicron (10 µg each animal) on day 700 after the first immunization. The sera were collected on day 700 and day 714. For immunization, groups of 6-8-week-old female BALB/c mice were immunized intramuscularly with RBD-Fc-Omicron vaccine (5 µg or 10 µg each mouse, n **=** 15 each group), or aluminum only on day 0, 14 and 28. Serum was collected at day 14, day 28 and day 42 post first immunization for detection of SARS-CoV-2-specific IgG and neutralizing antibody responses. Five mice were harvested on day 42 after the first immunization to evaluate the T cell responses.

Sera antibody titer evaluation

Specific IgG and isotype antibody titers were detected by using an ELISA method. Briefly, serial 2-fold dilutions of inactivated serum, starting at 1:100, were added to blocked 96-well plates (50 µl/well) coated with recombinant SARS-CoV-2 RBD (RBD-Omicron, RBD-WT, Sino Biological, Beijing, China) antigen and plates were incubated for 30 minutes at 37 °C. After three washes, plates were added with Horseradish peroxidase (HRP)-conjugated goat anti-mouse IgG (TransGen Biotech, Beijing, China)/IgG1/IgG2a/IgG2b (Abcam, Cambridge, UK) and incubated for 30 minutes at 37 °C, and then added with chromogen solution followed by 15 minutes of incubation at 37 °C. The absorbance (450/630 nm) was read using a microplate reader (BioTek, Vermont, USA). The endpoint titers were defined according to the manufacturer’s instruction.

Pseudovirus-based neutralization assay was performed as below. Serial 3-fold diluted serum, starting at 1:50, were incubated with 1024 TCID50 of the pseudovirus [pseudovirus carrying S protein of B.1.1.529.1 (Omicron BA.1), B.1.1.529.2 (Omicron BA.2), B.1.1.529.2.12.1 (Omicron BA.2.12.1), B.1.1.529.3 (Omicron BA.3), B.1.1.529.4 (Omicron BA.4), Delta variants (B.1.617.2, Delta 617) and B.1.640.2 (IHU), which were purchased from Tiantan Biological Products Co., Ltd., Beijing, China], and then Huh7 cells were seeded in 96-well plates (200,000 cells/well) and incubated at 37 °C for 1 hour. The supernatant was then removed and luciferase substrate (PE, Waltham, USA) was added to each well followed by incubation for 2 minutes in darkness at room temperature. Luciferase activity was then measured using GloMax® 96 Microplate Luminometer (Promega, Madison, USA).

A micro-neutralization assay was carried out to detect neutralizing antibodies against SARS-CoV-2 infection. Briefly, sera at 2-fold serial dilutions were incubated with 100 TCID50 SARS-CoV-2 (B.1.1.529.1, B.1.1.529.2), started at 1:2 or 1:100 dilution, for 1 h at 37 ℃, and then 50 μl (1.5×10^5^/ml) Vero cells were added to each well. The cells were observed daily for the presence or absence of virus-induced Cytopathic Effect (CPE) and recorded at 72 h. Neutralizing antibody titers were determined as the highest dilution of sera that can completely inhibit virus-induced CPE in 50% of the wells (NT50). The baseline of this assay was the 4-fold of assignment. In our study, the value of negative control was assigned to one.

Mice challenge studies

BALB/c mice were immunized with RBD-Fc-Omicron Vacc on day 0, day 14 and day 28. Two weeks after the last immunization, mice were inoculated with 3×10^3^ TCID50 Omicron BA.1 and BA.2 intranasally. On day 3 post infection, six mice in each group were sacrificed, and their lungs were removed for detection of viral load. This infection model is not lethal; thus, mice were euthanized with isoflurane overdose and the SARS-CoV-2 RNA levels were quantified in lungs by qRT-PCR to determine the virus loads with a pair of primers targeting N gene under standard curve method (Forward, GGGGAACTTCTCCTGCTAGAAT; Reverse, CAGACATTTTGCTCTCAAGCTG).

Histopathology assay

BALB/c mice were immunized with RBD-Fc-Omicron Vacc on day 0, day 14 and day 28. Two weeks after the last immunization, mice were inoculated with 3×10^3^ TCID50 Omicron BA.1 and BA.2 intranasally (6 mice each group). Three days post virus challenge, animals were sacrificed and lung tissues were obtained. Tissues were fixed in 10% neutral buffered formalin, embedded in paraffin, sectioned, and stained with hematoxylin and eosin. Lung tissue lesions were assessed according to the extent of denatured and collapsed bronchiole epithelial cells, degeneration of alveoli pneumocytes, infiltration of inflammatory cells, edema, hemorrhage, exudation and expansion of parenchymal wall.

Flow cytometry

Preparation of spleen single cells was described as below. In brief, cells isolated from spleen were incubated with Fc Block (clone 2.4G2) for 15 min at 4 ˚C, washed 3 times. For enumeration of spleen lymphocytes were stained on ice with anti-CD3-PerCP-eFlourTM-710 (clone17A2), anti-CD4-eFlour®450 (cloneRM4-5), anti-CD8a-PE-Cy7 (clone53-6.7) (eBioscience, Santiago, USA). Data were gated for forward scatter/side scatter and collected on an FACSCanto II (BD Biosciences, Franklin Lake, USA) and analyzed using FlowJo software (Tree Star).

Statistical analysis

Statistical analyses were performed using the program Prism 8.0 (GraphPad Software, Inc., La Jolla, USA). Values are expressed as mean ± SD. Data were analyzed by unpaired Student’s t-test (normal distribution) or one-way ANOVA followed by Dunnett’s multiple comparison test. P < 0.05 was considered to be statistically significant.


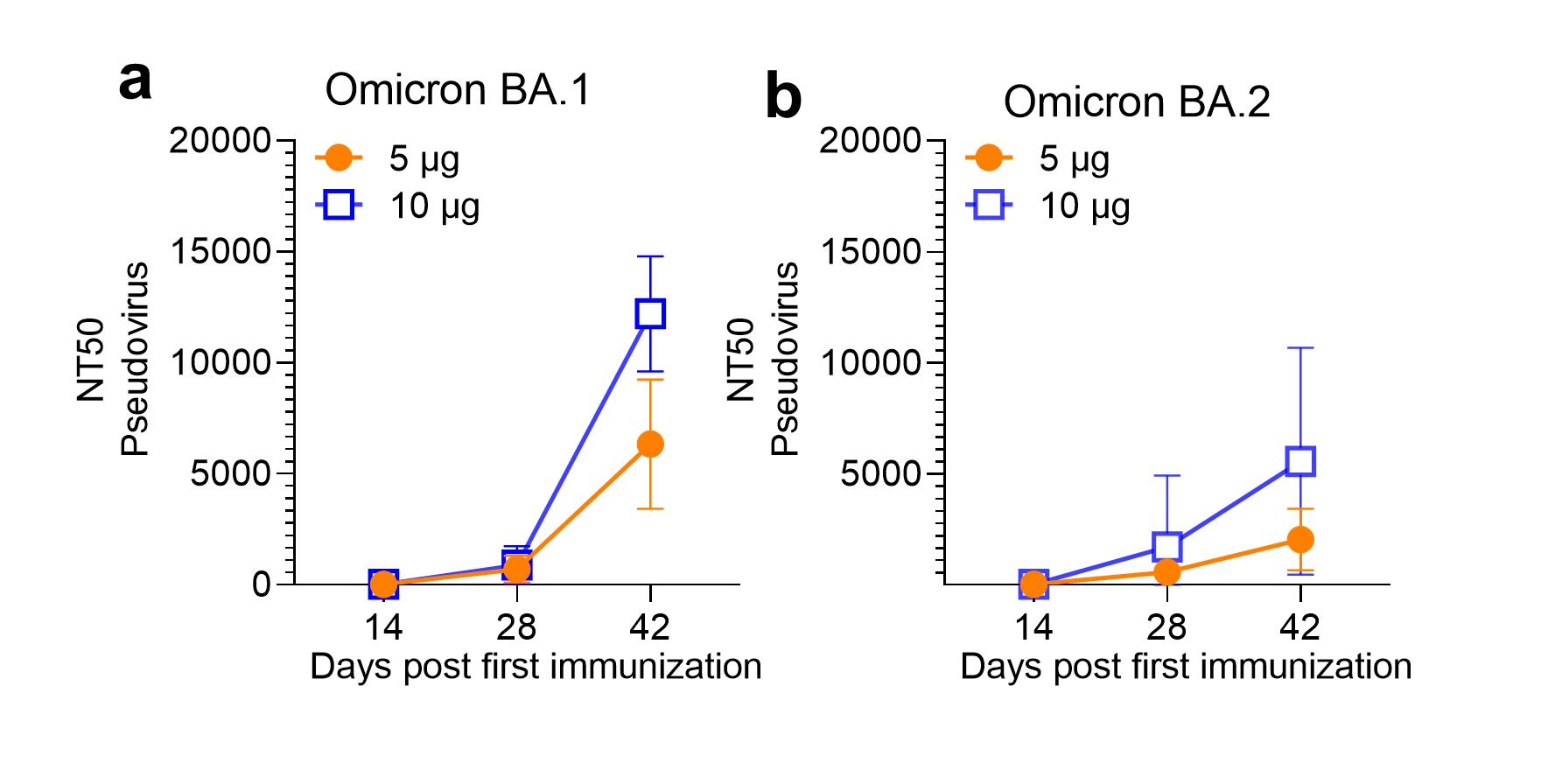


Figure. S1. Time course of neutralizing antibody titers against Omicron BA.1 and Omicron BA.2 after RBD-Fc-Omicron immunization. Groups of female BALB/c mice (n = 9-10) were immunized intramuscularly with three doses of 5 μg or 10 μg of RBD-Fc-Omicron at 14-day interval. Sera were collected on day 14, day 28 and 42 after the first immunization. The 50% neutralizing titers against pseudovirus Omicron BA.1 (a) and Omicron BA.2 (b) were analyzed.


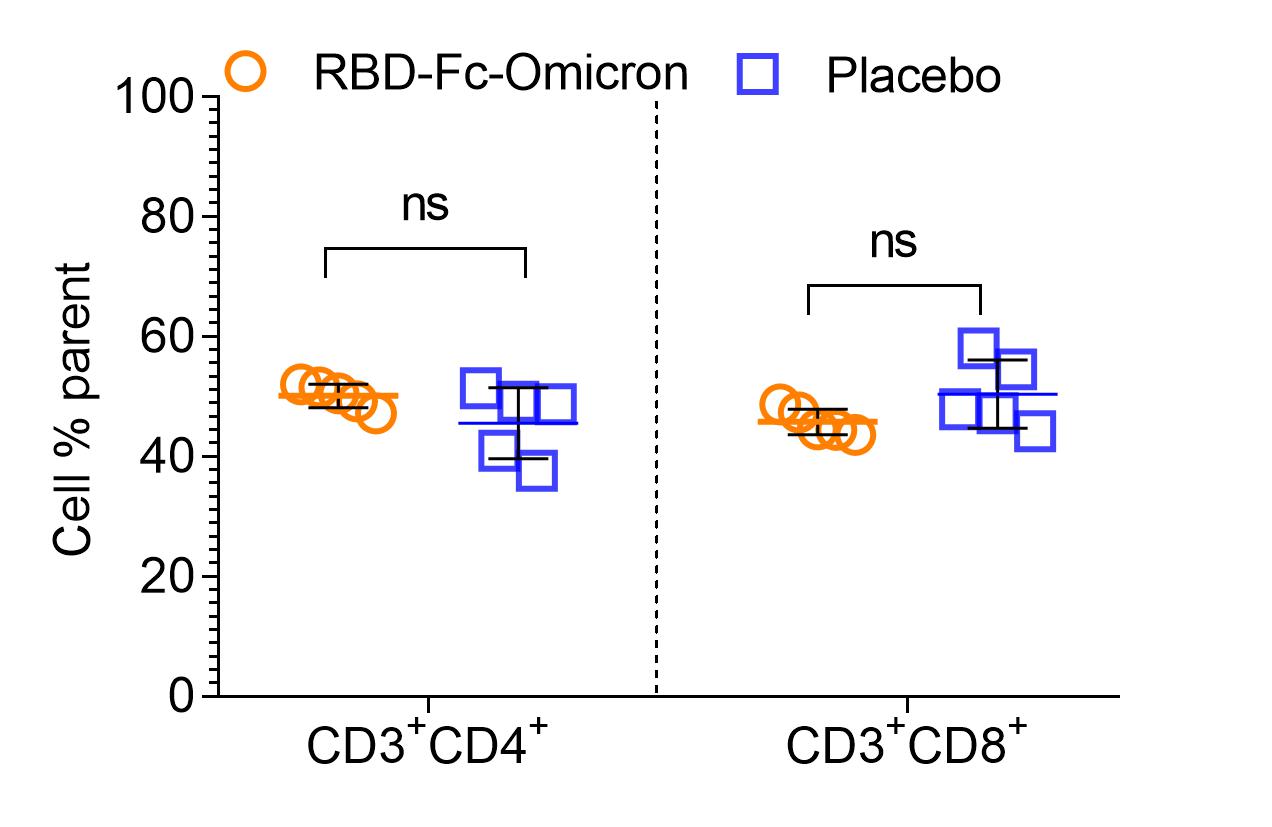


Figure. S2.T cell responses after RBD-Fc-Omicron immunization. Groups of female BALB/c mice (n = 5) were immunized intramuscularly with three doses of 10 μg of RBD-Fc-Omicron and placebo at 14-day interval. The spleen cells were harvest on day 42 after the first immunization. The flow-cytometer was used to evaluate the CD4^+^ T and CD8^+^ T cell responses. ns, no significant.
